# Moving towards a single-frame cell phone design in random digit dialing surveys: considerations from a French general population health survey

**DOI:** 10.1186/s12874-022-01573-1

**Published:** 2022-04-03

**Authors:** Noémie Soullier, Stéphane Legleye, Jean-Baptiste Richard

**Affiliations:** 1 Santé publique France, DATA, Unité Enquêtes, 12 rue du Val d’Osne, 94415 Saint-Maurice Cedex, France; 2 Insee, 88 avenue Verdier, 92120 Montrouge, France; 3 Equipe S-Pri, INSERM UMR-1178, CESP, Université Paris-Sud, Faculté de Médecine Paris-Sud, F-94276 Le Kremlin Bicêtre, France

**Keywords:** Survey, Sampling, Cell phone, Random digit dialing, Single-frame, Health

## Abstract

**Background:**

Over the last two decades, telephone surveys based on random digit dialing have developed considerably. At the same time, however, the proportion of the population with a cell phone has increased, whereas landline frame coverage has declined, thus raising the possibility of discontinuing landline phone surveys. This paper aims to assess the impact of using a single-frame (SF) cell phone design instead of a dual-frame (DF) design with landlines and cell phones in the context of repeated health surveillance surveys in the general population. We analyze data from a random digit dialing health survey of the French population and assess differences between the DF and the counterfactual SF design that excludes the landline phone sample from the DF design. We evaluate the quality of the two survey designs in terms of survey productivity, response rates, representativeness, balancing of external covariates, and prevalence estimates of key health behavior indicators.

**Results:**

Our results show that a SF cell phone survey has several advantages over a combined DF landline and cell phone survey. Cell numbers require fewer call attempts to complete an interview, leading to a substantial reduction in the mean data collection duration and weight dispersion. The global representativeness of the SF design was slightly better than its DF counterpart, although the elderly were underrepresented. After calibration, differences in health behavior estimates were small for the seven health indicators analyzed.

**Conclusions:**

Switching from a DF random telephone survey to a SF cell phone design has a number of practical advantages and would have a minimal impact on general population health surveys for monitoring health behavior at the population level. However, the different aspects of the survey quality had to be studied to make a decision. Further studies are needed to explore the scope of possibilities.

**Supplementary Information:**

The online version contains supplementary material available at 10.1186/s12874-022-01573-1.

## Background

Over the last two decades, telephone surveys based on random digit dialing (RDD) have developed considerably, especially in the field of general population health surveys. However, coverage error issues have also emerged in telephone surveys with the spread of cell phones and their progressive replacement of landlines. As a result, telephone survey designs have been adapted to include cell phone owners [1–4]. Dual-frame (DF) surveys, which include samples from both cell and landline telephones, have been growing in popularity, while the international literature on the methodological and practical challenges associated with these changes has developed considerably in recent years [5–14].

Several considerations could suggest to use cell phone only surveys. First, the equipment rate in cell phone is currently very high. Indeed, smartphones are now owned by about 80% of the population of the higher ranked western countries since 2017 (UK, Germany, USA, the Netherlands, Sweden, Belgium, France) (source Pew Research Center^1^). On the opposite, landline equipment, which was the norm few decades ago, has decreased continuously and is expected to continue decreasing even further in the coming years, as we observe the emergence of cell-phone only respondents, that is, individuals who never use their landline despite having an active number and who cannot be reached by landline sampling frames [15, 16]. In France, the last Information and Communication Technologies (ICT) survey conducted by the French national statistical institute [17] shows that in 2021, 99% of the population owns a telephone (be it a cell phone or a landline phone), 96% owns a cell phone and 77% of the population owns a landline telephone.

Furthermore, several well-identified limitations of cell phone interviews have diminished in recent years: poorer interview quality (poorer communication, shorter interview duration), higher cost per interview, lower response rates, higher refusal rates, and so on [15, 18]. With the growing experience in cell phone interviews, several benefits are even appearing. First, a cell phone sample offers the possibility of sending an SMS to each generated number, whereas for landline phone numbers, only addresses listed in the telephone directory can receive an invitation [19]. Other benefits derive from the fact that cell phones are personal equipment, thus allowing a single-stage design to directly interview the person who picked up the phone. By contrast, contacting individuals on landline phones implies a two-stage sampling design (sampling telephone numbers and then sampling a single respondent among the eligible persons in the household). Moreover, whereas respondents with both a landline and a cell phone may have different responses depending on the frame from which they are selected, less social desirability bias has been found with mobile phone interviews [20].

These changes impact the risk-benefit ratio of interviewing respondents on landline phones versus cell phones. Recent studies have assessed the feasibility of using a single-frame (SF) cell phone design that excludes the landline phone sample [21, 22]. In SF designs, it is easier to define a design weight, as each person can only be sampled one way. Also, a SF cell phone design means a single-stage design, which implies less variability of the design weights, thus resulting in a better accuracy of estimates. Additionally, this reduces the duration of the interview contact phase dedicated to the household screening. By contrast, a single-stage cell phone design does not cover people only equipped with landline telephone, which could lead to differences in sample representativeness compared to the DF design as well as differences in the survey estimates. While the aim of maximizing the population coverage rate continues to favor DF designs, recent results have suggested that moving to a SF cell phone design should be considered for public opinion surveys or subgroups such as young adults [22, 23].

Apart from the productivity gained from the use of cell phones, which is well known to polling organizations that conduct dual-frame telephone surveys, the abovementioned studies provide evidences in favor of a SF cell phone design but mostly in the context of the USA. Would these conclusions be valid in other cultural contexts? The differences in cell-phone equipment rates between the USA and most western European countries are quite small and decreasing over time (see above). But using cell phones to conduct surveys is still not as common in Europe as in the USA, the equipment rate by age is certainly not the same and many uncertainties remain. In France, according to the last ICT survey, cell phone equipment is not evenly distributed in the population: the equipment rate decreases with age (from 99% in the 15–29 years old to 80% in the 75 years old and more), and increases with educational degree (from 87% in those with primary education only to 96% in those with a secondary degree and more than 98% among those having at least 3 years of University). In France, excluding the landline numbers from the sampling frame may thus alter the estimates of specific health risk factors, as part of the elderly and the less educated and affluent people would be excluded of a cell phone only design. Moreover, regarding health topics, previous works have highlighted differences between landline and cell phone users in terms of health behaviors such as alcohol consumption, tobacco use or physical activity, and these differences were not totally explained by differences in socio-demographic characteristics taken into account in the weighting strategy [24–30]. The gain or loss in representativeness of the target population and the changes in outcome estimates of interest thus need a precise quantification. European health survey designers are eager to get conclusive evidences in their countries.

The aim of this article is to assess the impact of using a counterfactual SF cell phone design in the context of a repeated health surveillance survey in the general population whose sample was actually collected using a DF design. Several important issues need to be considered here: the sociodemographic characteristics of a cell phone sample among 18–75 year-olds and the representativeness of the reference population, weighting consequences, the impact on health estimators, and the accuracy of the results used to monitor public health policies. In particular, identifying whether this alternative design underrepresents particular groups is especially important, since health indicators and some risk factors are distributed unevenly across the socioeconomic gradient.

This analysis is conducted in terms of survey productivity including response rates, balancing of the sample in terms of calibration covariates (representativeness), balancing of external covariates, and prevalence estimates of key health indicators.

## Methods

The French Health Barometer is a repeated health telephone survey that has been conducted in the general French population since 1992 by Santé publique France, the national public health agency, allowing trends in health risk behaviors to be measured. To meet the challenge of adequately covering the population, the sampling design of the Health Barometer survey has regularly evolved. From 1992 to 2005, it used a SF directory-based survey of landline phone numbers. From 2005 to 2014, the survey used a screening approach with a disjoint DF sample of landline telephones and cell phone-only owners. Since 2014, an overlapping DF design of landline and cell phone numbers has been implemented using RDD [26]. Considering the recent evolutions in telephone usage, we question whether it is time to stop contacting people on landlines. It is therefore necessary to evaluate the impact of such a change on our estimates.

1. Health Barometer data and design weight

The 2017 French Health Barometer survey used the RDD method, and included a respondent sample of 25,319 people aged 18 to 75 years, living in France, non-institutionalized, and speaking French. Interviews were conducted using computer-assisted telephone interviewing. Participation was anonymous and voluntary. In accordance with the guidelines of the French Data Protection Authority (Commission Nationale de l’Informatique et des Libertés, CNIL), all subject included in this study gave their informed verbal consent to participate before the telephone interview.

The Health Barometer survey 2017 used an overlapping DF design of landline and cell phone numbers. For cell phones, the selected interviewee was the person who picked up the phone. For landline phones, the Kish selection method was used: it consists in listing all household members and then drawing randomly the person to interview among the eligible persons, with the same probability for each eligible person [31]. Thus, the sampling design was one-stage for cell interviews and two-stage for landline interviews. Hereafter, the sampling frame is the list of generated telephone numbers.

Design weights, reflecting the individual selection probability, were calculated for the DF sample (*df*) using information about the number of phone numbers generated, the number of phone numbers owned by the respondent (reported in the questionnaire), and the number of eligible persons in the household for landlines. Design weights are the inverse of the individual selection probability ${\pi}_i^{df}$, defined as:

$${\pi}_i^{df}=\frac{n_{LL}}{N_{LL}}\ast \frac{t_{LL}^i}{e_{LL}^i}+\frac{n_C}{N_C}\ast \frac{t_C^i}{e_C^i}$$

where *i* denotes an eligible person; *N*_*LL*_ and *N*_*C*_ denote, respectively, the number of landline and cell phone numbers in the sampling frame; *n*_*LL*_ and *n*_*C*_ denote, respectively, the number of landline and cell phone numbers in the sample; ${t}_{LL}^i$ and ${t}_C^i$ denote, respectively, the number of landline and cell phone numbers leading to contact with person *i*; ${e}_{LL}^i$ denotes the number of eligible persons per household for the landline number *i*, and ${e}_C^i$ denotes the number of eligible individuals who use the cell number $i\ \left({e}_C^i=1\right)$.

2. Counterfactual cell-phone only design weight

For the purpose of this study, we created an “as-if” SF cell phone sample (*sf*) by selecting cell phone respondents of our survey, i.e. by excluding the landline phone respondents from the DF sample. These weights, computed for the cell phone respondents only, are the inverse of ${\pi}_i^{sf}$, with:

$${\pi}_i^{sf}=\frac{n_C}{N_C}\ast \frac{t_C^i}{e_C^i}$$

3. Calibrated weights

The design weights (DF and SF) were then calibrated to adjust to the French population structure as reported by the Labor Force Survey (conducted by the French National Institute for Statistics and Economic Studies, INSEE) using the raking ratio [32]. The calibration covariates were gender by age, education level, size of household, urbanization, and region of residence. Note that these variables include age and education that are prominent determinants of health behaviors [33] (see Table 1 for details).

4. Impact of a SF cell phone design

**Table 1:** Distribution of calibration covariates in the reference population and standardized distances

	Reference population (%)	Dual-frame: Combined (d*)	Single-frame: Cell phone (d*)
***Sample size***		***25,319***	***15,602***
**Sex**		**5.6**	**3.0**
Male	48.7%	−5.6	−3.0
Female	51.3%	5.6	3.0
**Age (years)**		**2.0**	**2.7**
18–24	11.2%	−2.3	1.3
25–34	17.1%	0.5	5.2
35–44	18.5%	−2.3	0.8
45–54	19.5%	−1.5	−0.3
55–64	18.2%	2.6	0.5
65–75	15.5%	2.7	−8.1
**Education level**		**10.3**	**13.1**
Primary education	13.3%	−19.8	−22.9
Less than high school	35.4%	−8.9	−14.6
High school graduate	20.4%	3.2	5.6
2 years post-secondary	12.8%	−0.6	1.8
3–4 years post-secondary	7.6%	22.4	24.1
≥ 5 years post-secondary	10.5%	6.6	9.5
**Size of household**		**7.4**	**5.0**
1 person	17.4%	14.7	12.3
2 persons	34.2%	2.5	−2.3
3 persons	18.9%	−6.0	− 2.3
4 persons	19.3%	−8.4	−5.4
≥ 5 persons	10.2%	−5.5	−2.7
**Urbanization**		**2.6**	**2.5**
Rural	24.0%	2.3	−2.8
< 20,000 inhabitants	16.8%	−0.8	−1.9
20,000–99,999 inhabitants	12.1%	2.1	1.8
100,000–199,999 inhabitants	4.9%	4.4	4.8
≥ 200,000 inhabitants	25.7%	−4.2	−1.4
Paris agglomeration	16.6%	−1.9	2.1
**Region of residence**		**1.7**	**1.7**
Ile-de-France	19.0%	−2.8	1.6
Grand-Est	8.6%	−0.4	−1.9
Hauts-de-France	9.3%	−2.5	− 2.4
Normandie	5.1%	1.3	−0.8
Centre	4.0%	−1.0	−2.1
Bourgogne-Franche-Comté	4.4%	−0.5	−1.7
Bretagne	5.1%	3.9	3.2
Pays de Loire	5.7%	−0.9	−2.6
Nouvelle Aquitaine	9.2%	1.0	0.9
Auvergne-Rhône-Alpes	12.2%	3.0	1.8
Occitanie	9.1%	1.4	1.5
PACA and Corse	8.3%	−1.5	0.2
**Mean D**	**–**	**4.9**	**4.7**
**R-indicator without interactions**	**–**	**0.85**	**0.89**

Boldface entries correspond to results summed up at the variable level or to global distribution of the sample (mean D, R-indicator)

To evaluate the impact of moving to a SF cell phone design in terms of effectiveness and related costs, we first compared the performances of landline and cell phone interviews, using productivity criterions (interview duration, call attempts and generated phone numbers required to obtain an interview) and response rates. The original disposition codes used to calculate the response rates were mapped to the specified codes and formula #3 of the American Association for Public Opinion Research (AAPOR) (The American Association for Public Opinion Research, 2016, Survey Outcome Rate Calculator 4.0.)

Second, we studied the representativeness of the current dual-frame design (DF) and the counterfactual single-frame (SF) cell phone design, by using the corresponding design weights defined above. For this purpose, we studied first the balance of the effective DF and of the counterfactual SF samples to the population reference margins for each of the calibration covariates. The reference margins of the population for calibration covariates were taken from the 2016 Labor Force Survey conducted by INSEE, restricted to the same age-range (18–75 years old). The representativeness of each sample (DF and SF) was then also assessed for several external covariates using the corresponding calibrated weights. These external covariates were socio-demographic characteristics (occupational status and socio-professional group), and a health-related outcome (visits to a family physician in the preceding year), for which a gold-standard was available. These gold standards were taken from the 18–75 years old population in the 2016 Labor Force Survey for employment status and socio-professional group, and from National Health Insurance data for visits to a family physician in the preceding year. To compare samples in a univariate approach, we used standardized distances *d* [34]. For each variable category, a *d*_*category*_ was calculated as in formula (1) with *p*_*A*_ the prevalence in the reference population; *p*_*B*_ the weighted prevalence estimated in the sample; *qA* = 1 − *pA*; *qB* = 1 − *pB*. The information was then summed up at the variable level using a mean of absolute values of *d* on all *m* categories. Finally, at the sample level, we summed up the distances with a mean of all *D*_*variable*_. Following Austin’s recommendations [34, 35], we considered a value below 10% as reflecting an acceptable balance.

1 $${d}_{category}=100\times \left({p}_B-{p}_A\right) \left/ \sqrt{\left({p}_A{q}_A+{p}_B{q}_B\right)\left/ 2\right.}\right.$$

To provide a multivariate insight into the global distribution of a sample, we used R-indicators [36], which were based on a response propensity model. R-indicators estimate the variation of the predicted probability of belonging to the studied sample instead of the benchmark sample, conditionally to the variables included in the model. R varies between 0 and 1; the higher the value, the better the balance conditionally to all variables. We computed a model without interactions. The 2017 annual census, which collected data on 6,162,026 individuals aged 18–75 years living in non-institutionalized households, was used as the reference population to compute R-indicators.

In a third step, we analyzed the impact of switching to a SF cell phone design for seven health indicators: self-reported health status as “poor,” chronic diseases, limitations in daily activities, obesity (body mass index ≥30), physical inactivity (physical activity of at least 30 min less than once a month), daily cigarette smoking, and lifetime suicidal attempts. The first three indicators compose the Minimum European Health Module (MEHM), which is a standardized instrument used to monitor the different dimensions of health at national and international levels [37]. For the mental health dimension, we chose lifetime suicidal attempts as France has one of the highest suicide rates in Europe [38]. Finally, tobacco smoking and obesity were studied as important risk factors for a number of non-communicable diseases, including cancer, diabetes and cardiovascular disease [39, 40]. These risk factors were described in previous studies to be inequitably distributed across the different socio-economic strata, that is obesity and tobacco smoking are concentrated in the least affluent and the least educated parts of the population, contributing to increase the social inequalities in health [41–43].

We provided prevalence estimates calculated for the combined DF sample and the counterfactual SF sample using the corresponding calibrated weights. The health indicators were estimated for the whole population, and also for two age subgroups (18–30 year-olds and 60–75 year-olds) being respectively the most and the least equipped with cell-phones. To quantify the possible impact on the prevalence estimates, we used the relative difference between the combined DF sample $({\overline{y}}_{df})$ and the SF sample $({\overline{y}}_{sf})$, defined as:

$$Relative\ Difference=\frac{{\overline{y}}_{sf}-{\overline{y}}_{df}}{{\overline{y}}_{df}}$$

The sampling design impact was evaluated using the *deft* indicator, defined as the square root of the design effect for each health indicator:

$$deft=\sqrt{\frac{\mathit{\operatorname{var}}\left(\overline{y}\right)}{s^2\left/ n\right.}}$$

where $\mathit{\operatorname{var}}\left(\overline{y}\right)$ is the variance observed for *y* on the sample, *s*^2^ is the variance estimated under a simple random sampling, and *n* is the sample size [44]. *s*^2^ equals *p(1-p)* when *y* is a binary variable of proportion *p*. *Deft* shows how much the survey design impacts the sample standard error (SE), and consequently, the confidence intervals.

Calibrated weights were also compared between the combined DF sample and the SF sample using the max/min ratio and the coefficient of variation, calculated as the ratio of the standard error over the mean.

Analyses were conducted using SAS 9.4 and Stata 14.2. Syntax used in the data analysis are provided in Supplementary materials. The data are available on request from the corresponding authors.

## Results

1. Performances of landline and cell phone interviews

The average survey duration was similar for both landline and cell phone interviews (31.3 min and 32.8 min, respectively). The longer contact phase for the landline interviews (due to the thorough screening for the Kish selection) was offset by the longer questionnaire for the cell phone interviews (due to the younger age of the cell phone respondents to whom more questions were asked, essentially relating to tobacco and alcohol consumption). The contact and screening phases were on average 2.3 min shorter in the cell phone interviews.

The average number of calls required to obtain a complete interview was also similar between the samples (9.0 for cell phone interviews vs 9.5 for landline interviews). However, contacting younger respondents (18–34 years) required fewer call attempts to cell phones on average (9.6 vs 11.9). This difference was less pronounced for 35–54 years-old (9.4 vs 10.6 call attempts) and reversed for 55–75 year-olds (8.3 vs 7.9 call attempts).

The productivity of telephone numbers also favored cell phones: on average, 16.5 landline phone numbers were needed to complete an interview vs 6.4 for cell phone numbers. Three reasons may explain this result: the higher percentage of non-working numbers in the landline frame (50% vs 33%), the two-stage sampling design on landline phones which could require another call to reach the interviewee selected by the Kish method if it is not the one who picked up the phone and if this person is not available, and the fact that people are close to their cell phone most of the time, as opposed to their landline phone. Indeed, 68% of the landline interviews were made among a household with more than one eligible person, and 31% of the landline interviews were not made with the person who picked up the phone.

Response rates were quite similar between landline and cell phone frames, being 37.8 and 34.8%, respectively, according to the AAPOR formula #3 (36.6% overall).

For more details on the performances of the landline and cell phone frames, see Supplementary Table 1 in the Supplementary Material.

2. Balance of calibration covariates (Table 1)

Regarding the standardized distances to the reference population using design weights, we observed shorter distances for the combined DF sample for age (especially for the elderly) and educational level with the combination of landline and cell phone profiles. On the other hand, the SF sample has quite shorter distances regarding sex and size of household. The combined DF sample and the SF sample had similar profiles, with the latter being slightly better overall (D = 4.7 vs 4.9). Some populations were underrepresented in both samples, such as people with primary education and households with more than three persons.

R-indicators corroborate these results, with a slightly better value for the SF sample compared to the DF sample.

3. Balance of external covariates (Table 2)

**Table 2:** Prevalence estimates and standardized distances to the reference population for external covariates

	Reference population (%)	Dual-frame: combined (d*)	Single-frame: cell phone (d*)
*Sample size*		*25,319*	*15,602*
**Professional status (Labor Survey) (1)**		**8.0**	**7.8**
Employed	58.7%	−6.1	−4.3
Student	5.0%	9.2	8.0
Unemployed	6.2%	11.6	12.0
Retired/inactive	30.2%	−5.3	−6.9
**Socio-professional group (Labor Survey) (2)**		**2.9**	**2.8**
Farmer	1.7%	0.8	0.8
Craftsperson, tradesperson, business owner	6.3%	0.0	2.4
Executive	17.2%	−7.1	−6.3
Intermediate occupations	25.1%	−1.9	−1.9
Technicians	27.9%	3.1	2.7
Blue-collar worker	21.8%	4.5	3.1
**Visit to a family physician in the last year (Health Insurance) (3)**		**6.6**	**8.2**
Yes	85.5%	−6.6	−8.2
No	14.5%	6.6	8.2
**Mean D (1) (2) (3)**	–	**5.8**	**6.3**

Boldface entries correspond to results summed up at the variable level or to global distribution of the sample (mean D, R-indicator)

Using calibrated weights for the combined DF, the combined DF sample and the SF sample had similar profiles regarding professional status and socio-professional group, with an overrepresentation of unemployed people and a small underrepresentation of executives.

The estimate for visiting a family physician in the preceding year was slightly closer to the health insurance estimate in the combined DF sample than in the SF sample (D = 6.6 vs 8.2).

Overall, the combined DF sample and the SF sample had good and similar D values for the external covariates.

4. Impact on estimates of health behaviors

Differences between the combined DF sample and the SF sample were rather small on the seven health indicators studied here, after calibrating each sample to sociodemographic covariates (Table 3). The absolute differences for the health behavior estimates between the combined DF sample and the SF sample do not exceed 1 percentage point, with the maximum difference being 0.6 for daily smoking (Table 3). The relative differences are up to  -5.0% for self-reported health status (minimum  -0.8% for chronic diseases). Differences were greater for the estimations according to age group, with  -11.9% relative difference for physical inactivity among younger respondents (Table 4), and + 8.7% relative difference for daily smoking among elderly respondents (Table 5). However, this corresponds to rather minor absolute differences: 0.8 and 1.1 percentage points, respectively.

**Table 3:** Health behavior estimates and design effect in the combined dual-frame sample and the single-frame sample

	Dual-frame: combined (*n* = 25,319)		Single-frame: cell (*n* = 15,602)		Relative difference
	*% (SE)*	*DEFT*	*% (SE)*	*DEFT*	*%*
Self-reported health status as “poor”	6.0 (0.2)	1.30	5.7 (0.2)	1.21	−5.0
Chronic diseases	36.6 (0.4)	1.22	36.3 (0.4)	1.13	−0.8
Limitations in daily activities	21.6 (0.3)	1.23	21.1 (0.4)	1.15	−2.4
Obesity	13.5 (0.3)	1.27	13.1 (0.3)	1.17	−3.2
Physical inactivity	8.7 (0.2)	1.32	8.9 (0.3)	1.20	2.0
Daily cigarette smoking	27.0 (0.4)	1.28	27.6 (0.4)	1.14	2.4
Lifetime suicidal attempt	7.2 (0.2)	1.28	7.4 (0.2)	1.15	2.4

Analyses used corresponding calibrated weights for each frame (combined dual-frame and single-frame)

**Table 4:** Health behavior estimates in the combined dual-frame sample and the single-frame sample for 18–30 year-olds

	Dual-frame: combined (*n* = 4452)	Single-frame: cell (*n* = 3503)	Relative difference
	*% (SE)*	*% (SE)*	*%*
Self-reported health status as “poor”	1.9 (0.3)	2.1 (0.3)	7.8
Chronic diseases	20.8 (0.7)	22.1 (0.8)	6.0
Limitations in daily activities	10.6 (0.6)	10.9 (0.6)	3.0
Obesity	6.9 (0.5)	7.2 (0.5)	4.3
Physical inactivity	6.9 (0.5)	6.1 (0.5)	−11.9
Daily cigarette smoking	34.5 (0.9)	35.5 (0.9)	2.9
Lifetime suicidal attempt	6.4 (0.5)	6.7 (0.5)	3.9

Analyses used proper corresponding weights for each frame (combined dual-frame and single-frame)

**Table 5:** Health behavior estimates in the combined dual-frame sample and the single-frame sample for 60–75 year-olds

	Dual-frame: combined (*n* = 7226)	Single-frame: cell (*n* = 3161)	Relative difference
	*% (SE)*	*% (SE)*	*%*
Self-reported health status as “poor”	8.7 (0.4)	8.1 (0.6)	−6.7
Chronic diseases	52.9 (0.7)	52.5 (1.0)	−0.9
Limitations in daily activities	31.2 (0.7)	30.1 (0.9)	−3.5
Obesity	18.2 (0.6)	16.8 (0.8)	−7.9
Physical inactivity	8.9 (0.4)	9.5 (0.6)	5.9
Daily cigarette smoking	12.3 (0.5)	13.4 (0.7)	8.7
Lifetime suicidal attempt	6.2 (0.3)	6.7 (0.5)	8.2

Analyses used corresponding weights for each frame (combined dual-frame and single-frame)

The design effect (*deft*) is always smaller for the SF sample (Table 3). The mean *deft* was 1.27 for the combined DF sample and 1.16 for the SF sample. Similarly, the coefficient of variation of the calibrated weights was smaller for the SF sample compared to the combined DF sample (0.51 vs 0.71), as was the max/min ratio (36.81 and 116.24, respectively).

Multivariate analyses comparing cell phone and landline phone respondents of the DF sample for the seven health indicators are provided in the Supplementary material.

## Discussion

Our analysis provides original results that complement the few studies that have examined the impact of moving from a combined dual-frame (DF) design to a single-frame (SF) cell phone design for general population surveys using RDD [21, 22]. This approach uses a large cross-sectional health survey, relies on gold standards to fully scrutinize differences in representativeness, and assesses this representativeness with appropriate tools such as the standardized distance and R-indicator.

Regarding the mean standardized distance, the combined DF sample and the SF sample have very similar profiles, with a slightly better global representativeness for the SF design. However, looking further into the detail, the combined DF sample is closest to the reference population with regard to age and education level. Indeed, the SF sample underrepresents the elderly (> 65 years old) and the low educated. Despite a steady increase in the use of cell phone equipment during the last few decades, the elderly remain only partially equipped, as only three-quarters of the population aged 65 years and over have a cell phone [45].

After calibration, the combined DF sample and the SF sample present similar values concerning the distances to external covariates: unemployed people are overrepresented in both designs. The SF sample has a lower distance to socioeconomic references such as professional status, socio-professional group, and landline phone equipment, but a greater distance concerning administrative health data. Individuals who visited their family physician during the last year are slightly underrepresented in the SF sample, suggesting that a different selection bias or a measurement bias may exist among cell phone respondents.

Overall, differences in health behavior estimates are rather small at the population level, being less than 0.6 percentage points or 5% of relative difference among the seven health indicators analyzed here. These results support the fact that moving to a SF cell phone design would have a minimal impact on public health messages issued from health surveys, as has already been concluded for public opinion surveys or youth risk behavior surveillance [22, 23]. However, some differences still exist. Cell phone respondents more often tended to be daily smokers and to report lifetime suicidal attempts (for older respondents), even after controlling for demographic characteristics. These results are consistent with those observed in other studies [14, 30, 46]. As a consequence, a small difference in the estimate of the proportion of daily smokers is observed between the combined DF sample and the SF sample (+ 0.6 point of prevalence). However, this difference is precisely in the significance area for the trend estimation of daily smoking, which is important since one of the main goals of this survey is to annually monitor tobacco consumption indicators and evaluate public policies.

Response rates, interview duration, and costs were similar in the cell phone and landline frames. However, cell numbers required fewer call attempts to complete an interview. Furthermore, as cell phones are considered to be personal equipment, no specific questions on household composition were necessary to correctly weight the questionnaire, thus leading to a substantial reduction in the mean duration of the interview (> 2 min). This one-stage sampling contributes to a reduction in the variance of the weights and thus to greater precision This could represent a gain in terms of precision or cost, as fewer interviews could be conducted for the same level of precision.

To summarize, RDD conducted with only cell phones has several advantages over a combined DF landline and cell phone survey: shorter questionnaire duration, higher productivity, and greater precision. In addition, because cell phones are personal devices, response rates are more accurate.

Our analysis uses data back from 2017. However, phone equipment in France has not evolved much since then, with 84% equipped with landline phone in 2020 (vs 86% in 2017) and 94% equipped with cell phone (constant since 2017) [45]. The last figures obtained by the national institute for statistics -with a more robust methodology- in 2021 are 96% of the population equipped with a cellphone (77% with a smartphone) and 77% equipped with a landline phone. In this most recent years, the landline phone equipment has continued to decrease for the younger ones (65% of the 18–24 years old in 2020 vs 81% in 2017) as the one of the cell phone has increased for the older ones (84% of the 70 years old and older in 2020 vs 76% in 2017), thus reinforcing the coverage of a survey that would be conducted only on cell phone numbers. This trend should reduce differences between the DF and the cell phone SF designs. The other main change in this recent years is the increasing use of a smartphone (84% vs 73% in 2017), especially for the elderly and the low educated, opening the way to new designs involving the smartphone as a way to interact with interviewees.

The question of switching to a single-frame design was here examined using an “as-if” counterfactual scenario from a dual-frame sampled survey. Thus, the single-frame respondents’ sample is a subsample of the dual-frame respondents’ sample and the two samples are not independent, preventing from testing differences statistically. Thus, differences could be underestimated. However, even in this conservative scenario, differences are revealed, especially in subgroups estimations.

One question that remains concerns the evaluation of the measurement bias between cell and landline phones. It was previously suggested that social desirability bias may increase in non-private situations and that cell phones could offer more opportunities to respond to the questionnaire in a private setting, as people can choose whether to answer the call and then where to go to continue the conversation after answering [47]. Despite multivariate adjustments (with age in a polynomial form), cell phone respondents showed specific differences compared to landline phone respondents (essentially for tobacco smoking and past suicidal attempts). These differences were more pronounced among the youth and the elderly. This suggests either a selection bias on unobservable characteristics (not included in the modeling) or a genuine measurement bias due to the interview context among others [47]. Disentangling the two is beyond the scope of this paper.

The dual-frame sampling was based on a stratification of 40% landline interviews and 60% cell phone interviews. As reliable estimates of the population’s equipment and uses was not available in France at that time, these proportions were assessed jointly with the survey institute but might differ from reality. Clearly, an optimal initial allocation [48] or reweighting would have reduced the variance of the estimates; however, the gain in variance resulting from the exclusive use of cell phones is certain and no optimal allocation could compensate for it. Exploring the sensitivity of our results to variations of the proportions of mobile and landline telephone is beyond the scope of this paper. However, beside the gain in variance, one may also consider the gain in bias and in representativeness.

First, one way to improve the representativeness of the respondent sample would be to use external data relating to telephone equipment and usage as well as health data in the weighting procedure. Regarding health, obtaining the true number of medical consultations for the reference population is challenging, because of the long duration of the data collection (9 months). In addition, this requires the accurate self-reporting of health variables by respondents, even though they may be subject to memory biases. Self-reporting about telephone equipment and usage are probably more accurate in comparison. National ICT surveys will provide soon the proportion of individuals equipped with a landline, cell phone, or smartphone, and their propensity to answer a call on each type of telephone. As the type of telephone is still linked to health behaviours, this is promising. Second, because there is a measurement bias when comparing answers obtained through mobile and landline telephone, the choice of the allocation of mobile and landline telephone in the sampling or reweighting design may also consider reference values for the variables of interest (obtained through counterfactual modelling or a reference survey).

Some individuals only own a landline telephone: no weighting procedure can correct for this non-coverage, which exposes phone surveys to a potential non-response bias if these non-respondents differ from respondents. Finding an adapted approach to adjust for phoneless and landline-only households in public health research is thus at stake [49]. The relevance of such weighting depends on the goal of the survey (trend monitoring or exact point estimates) as well as the variables and topics. For this purpose, control samples obtained using different data collection methodology would provide external benchmarks on key variables, which could be used in the weighting procedure, provided that the data collection method does not produce measurement biases [50, 51].

Finally, in France, only landline numbers from agreeing owners are recorded in the telephone directory, in which only 25% of households are effectively present. As a consequence, letters can only be sent in advance to these households, although this approach substantially enhances the response rate [52–54]. In addition, in France as in some other countries, a legal disposition allows any telephone owner to register in a file that prevents any commercial use of his number. Consequently, calls tend to be less accepted when the number is unknown: a substantial proportion of the cell phone owners never answers incoming calls from an unknown number, and in addition it is very easy to ban numbers with these devices. In France in 2021, according to the last ICT survey, 27% of the people owning exclusively a cell phone state that they systematically refuse incoming calls or filter those from an unknown number; this is also the case of 50% of the people owning exclusively a landline (compared to 16.5% of those owning the two). Pre-notification short text messages are also effective in improving participation [19], although the length of the message is usually set to the standard 160 characters (including spaces). New perspectives are now emerging, as a greater proportion of the population owns a smartphone. This technological support would allow a more detailed message to be sent in advance, although conducting rigorous experiments to assess the efficacy of such electronic advance letters is still required.

One remaining specificity of France is that it appears to be relatively unaffected by the ongoing decline in survey response rates compared to most countries in Europe or the United States [55]: response rates of probability dual-frame telephone surveys commonly exceed 45% [56, 57] and even reaches 70% in the Survey of Consumer Attitudes of the National Institute of Statistics [58] compared to about 16% in the USA (Dutwin and Lavrakas, 2017).

## Conclusion

Switching from a dual-frame landline and cell phone random telephone survey to a single-frame cell phone design has a number of practical advantages. However, the different aspects of the survey quality had to be studied to make a decision. In terms of sociodemographic characteristics of the respondents’ sample, the dual-frame includes more low educated respondents, more old respondents as well as more individuals from rural areas, leading to a slight better representativeness when considering studying such subpopulations. On the other hand, as less social desirability is expected on cell-phone answers, the single-frame could provide estimations closer to the real measure. Finally, the importance of evaluating trends over time should be considered, as the change of sampling frame would inevitably lead to a break in series. Further studies are needed to explore the scope of possibilities.

## Supplementary Information

**Additional file 1 : Supplementary Table 1.** Call outcomes, interview characteristics, and rates according to landlines, cell phones, and all types for the dual-frame sample. **Supplementary Table 2.** Health behavior estimates for landline and cell phone respondents in the Health Barometer survey 2017.

**Additional file 2.** Names of variables.

## Data Availability

The datasets used and/or analyzed during the current study are available from the corresponding author on reasonable request.

## Abbreviations

RDD: Random digit dialing
DF: Dual-frame
SF: Single-frame

## Footnotes

1 https://www.pewresearch.org/global/2019/02/05/smartphone-ownership-is-growing-rapidly-around-the-world-but-not-always-equally/.
