# Supplementary material for: Moving towards a single-frame cell phone design in random digit dialing surveys: considerations from a French general population health survey
Source: BMC Med Res Methodol. 2022 Apr 3;22:94. doi: 10.1186/s12874-022-01573-1 (PMC8978421; doi:10.1186/s12874-022-01573-1)
Supplement: Supplementary file 1 — Additional file 1 : Supplementary Table 1. Call outcomes, interview characteristics, and rates according to landlines, cell phones, and all types for the dual-frame sample. Supplementary Table 2. Health behavior estimates for landline and cell phone respondents in the Health Barometer survey 2017. [file 12874_2022_1573_MOESM1_ESM.docx]

**SUPPLEMENTARY MATERIAL**

Supplementary table 1. Call outcomes, interview characteristics, and rates according to landlines, cell phones, and all types for the dual-frame sample

|  |  | **Landline** | **Cell** | **Overall** |
| --- | --- | --- | --- | --- |
| T = Total phone numbers used | | 160,790 | 100,565 | 261,355 |
| I = Complete interviews (1.1) | | 9,717 | 15,602 | 25,319 |
| P = Partial interviews (1.2) | | - | - | - |
| R = Refusal and break-off with eligible case (2.1) | | 2,196 | 693 | 2,889 |
| NC = Non-contact with eligible case (2.2) | | 1,598 | - | 1,598 |
| O = Other non-interview with eligible case (2.0, 2.3) | | 420 | 602 | 1,022 |
| UH = Unknown if residential (3.0, 3.1) | | 21,324 | 17,840 | 39,164 |
| UO = Unknown other (3.2, 3.9) (residential, unknown if eligible) | | 11,248 | 25,018 | 36,266 |
| INNR = Ineligible: Non-residential (4.0,4.1,4.2,4.3,4.4,4.5,4.8,4.9) | | 109,462 | 36,581 | 146,043 |
|  | *Fax line* | *2,595* | *829* | *3,424* |
|  | *Non-working numbers* | *81,120* | *32,884* | *114,004* |
|  | *Business, government office, other organizations (INNRb)* | *25,747* | *2,868* | *28,615* |
| INR = Ineligible: Residential but ineligible for survey (4.7) | | 4,823 | 4,228 | 9,051 |
| e1 = % of known residential cases estimated to have eligible respondents | | 74% | 80% | 77% |
| e2 = % of unknown-if-residential cases estimated to be residential | | 22% | 56% | 34% |
| **Response rate** | |  |  |  |
| Response Rate 3: I / (I+P+R+NC+O+[e1*e2*UH]+[e1*UO]) | | 37.8% | 34.8% | 36.6% |
| **Survey duration and productivity** | |  |  |  |
| Average survey duration in minutes (median) | | 31.3 (29.8) | 32.8 (30.7) | 32.2 (30.4) |
|  | *Average contact and screening phase duration in minutes (median)* | *4.7 (4.4)* | *2.4 (2.3)* | *3.2 (3,1)* |
| Average number of calls required before interview (median) | | 9.5 (5.7) | 9.0 (5.4) | 9.2 (5.5) |
| Telephone numbers used to obtain a contact: T/(I+R+INR+INNRb) | | 3.8 | 4.3 | 4.0 |
| Telephone numbers used to obtain an eligible contact: T/(I+R) | | 13.5 | 6.2 | 9.3 |
| Telephone numbers to obtain a complete interview: T/I | | 16.5 | 6.4 | 10.3 |

Supplementary table 2. Health behavior estimates for landline and cell phone respondents in the Health Barometer survey 2017

|  | Dual-frame:  landline (n=9,717) | Dual-frame:  cell  (n=15,602) | Crude RR^1^ (ref=landline)  (n_LL_=9,717; n_CL_=15,602) | Adjusted RR^2^  (ref=landline)  (n_LL_=9,717; n_CL_=15,602) | 18-30 year-olds adjusted RR^2^  (ref=landline)  (n_LL_=949; n_CL_=3,503) | 60-75 year-olds adjusted RR^2^  (ref=landline)  (n_LL_=4,065; n_CL_=3,161) |
| --- | --- | --- | --- | --- | --- | --- |
| Self-reported health status as “poor” | 6.6% | 5.7% | 0.86* [0.76; 0.98] | 1.00 [0.88; 1.14] | 1.10 [0.56; 2.15] | 1.08 [0.89; 1.31] |
| Chronic diseases | 41.4% | 33.8% | 0.82* [0.79; 0.85] | 0.98 [0.95; 1.02] | 1.12 [0.94; 1.33] | 1.00 [0.95; 1.06] |
| Limitations in daily activities | 24.8% | 19.8% | 0.80* [0.76; 0.85] | 0.98 [0.92; 1.03] | 1.12 [0.86; 1.46] | 0.98 [0.90; 1.06] |
| Obesity | 15.7% | 12.3% | 0.79* [0.73; 0.85] | 0.93 [0.86; 1.01] | 0.96 [0.70; 1.31] | 0.95 [0.84; 1.08] |
| Physical inactivity | 8.3% | 9.0% | 1.09 [0.98; 1.21] | 1.11 [0.99; 1.25] | 0.74 [0.54; 1.01] | 1.19 [0.98; 1.43] |
| Daily cigarette smoking | 20.3% | 30.7% | 1.51* [1.43; 1.60] | 1.15* [1.09; 1.22] | 1.32* [1.15; 1.51] | 1.21* [1.05; 1.41] |
| Lifetime suicidal attempt | 6.5% | 7.7% | 1.19* [1.06; 1.34] | 1.07 [0.94; 1.21] | 1.04 [0.73; 1.49] | 1.35* [1.09; 1.68] |
| *All analyses used calibrated weights for the combined dual-frame sample.*  ** p-value < 0.05*  *^1^ Risk ratio for Poisson regression model with robust variance estimation*  *^2^ Risk ratio for Poisson regression model with robust variance estimation adjusted for sex, age (polynomial), education level, urbanization, size of household, region of residence, employment status, socio-professional group, and level of income (tertiles)* | | | | | | |
