# Supplementary material for: Moving towards a single-frame cell phone design in random digit dialing surveys: considerations from a French general population health survey
Source: BMC Med Res Methodol. 2022 Apr 3;22:94. doi: 10.1186/s12874-022-01573-1 (PMC8978421; doi:10.1186/s12874-022-01573-1)
Supplement: Supplementary file 2 — Additional file 2. Names of variables. [file 12874_2022_1573_MOESM2_ESM.docx]

**Names of variables**

Individual weights for the Dual-frame (combined) : **poids_ind**

Individual weights for the Single-frame (cellular) : **poids_ind_mobile**

Calibration covariates : **sexe age_6 DIPLOME TAILLE_FOYER CAT_AGG6 region_calage**

Landline frame or cellular frame : **fixmob**

**SAS codes for R-indicator**

%macro r_indic(input=, ass=, model=) ;

proc delete data=outr ; run ;

ods select output ;

ods select association SelectedEffects ;*=&ass.;

title "&input., &model." ;

proc hplogistic data=&input.;

class sexe age6cl situat_r diplome region tuu nbpers_r sitprof4 gs4 ;

model bs(ref='0')= &model./ rsquare lackfit association ;

weight pnorm ;

output out=outr predicted=ps ;

run ;

ods select all ;

proc means data=outr std ;  var ps ;run ;

title ;

%mend ;

/*SIMPLE R*/

**%r_indic(input=bsrrp, ass=DUALFRAME, model=sexe age6cl diplome region tuu nbpers_r) ;**

/*R with all bivariate interactions*/

**%r_indic(input=bsrrp, ass=SINGLEFRAMECELL, model=sexe|age6cl|diplome||region|tuu|nbpers_r  @2) ;**

**SAS codes for Table 1 and Table 2**

**Dual-frame : combined**

**proc** **surveyfreq** data=bs2017_fm;

tables sexe age_6 DIPLOME TAILLE_FOYER CAT_AGG6 region_calage /nowt nototal;

weight poids_ind;

**run**;

**Single-frame : cellular**

**proc** **surveyfreq** data=bs2017_fm;

tables sexe age_6 DIPLOME TAILLE_FOYER CAT_AGG6 region_calage /nowt nototal;

weight poids_ind_mobile;

**run**;

**SAS codes for Table 3a, 3b, 3c**

**Example for Obesity (same code for other health behaviors)**

**Dual-frame : combined**

**proc** **surveyfreq** data=bs2017_fm ;

tables obesite/ nowt cl(type=cp) ;

weight poids_ind;

**run**;

**proc** **surveyfreq** data=bs2017_fm ;

tables obesite/ nowt cl(type=cp) ;

where **18**<=age<=**30**;

weight poids_ind;

**run**;

**proc** **surveyfreq** data=bs2017_fm ;

tables obesite/ nowt cl(type=cp) ;

where 60<=age<=75;

weight poids_ind;

**run**;

**Single-frame : cellular**

**proc** **surveyfreq** data=bs2017_fm ;

tables obesite/ nowt cl(type=cp) ;

weight poids_ind_mobile;

**run**;

**proc** **surveyfreq** data=bs2017_fm ;

tables obesite/ nowt cl(type=cp) ;

where **18**<=age<=**30**;

weight poids_ind_mobile;

**run**;

**proc** **surveyfreq** data=bs2017_fm ;

tables obesite/ nowt cl(type=cp) ;

where 60<=age<=75;

weight poids_ind_mobile;

**run**;

**SAS codes for Table 5 : Dual-frame: landline and Dual-frame: Cellular estimates**

**Example for Obesity (same code for other health behaviors)**

**proc** **surveyfreq** data=bs2017_fm;

tables obesite*fixmob/col nowt nostd nototal nocellpercent chisq;

weight poids_ind;

**run**;

**Stata codes for Table 5 : Poisson regression models**

**Example for Obesity (same code for other health behaviors)**

**xi:poisson obese_bin i.fixmob [pweight=poids_ind], vce(robust) irr compare**

**/*all : polynomial form for age*/**

**xi:mfp:poisson obese_bin age i.sexe i.cat_agg6 i.diplome i.taille_foyer i.rev_3k i.sitprof i.pcs7 i.region_calage i.fixmob [pweight=poids_ind], vce(robust) irr compare**

testparm Iage__*

testparm _Icat_agg6_*

testparm _Idiplome_*

testparm _Itaille_fo_*

testparm _Irev_3k_*

testparm _Isitprof_*

testparm _Ipcs7_*

testparm _Iregion_ca_*

**/*18-30 years */**

**xi:mfp:poisson obese_bin age i.sexe i.cat_agg6 i.diplome i.taille_foyer i.rev_3k /*i.sitprof*/ i.pcs7 i.region_calage i.fixmob if age >=18 & age<=30 [pweight=poids_ind], vce(robust) irr compare**

**/*60-75y : polynomial form for age*/**

**xi:mfp:poisson obese_bin age i.sexe i.cat_agg6 i.diplome i.taille_foyer i.rev_3k /*i.sitprof*/ i.pcs7 i.region_calage i.fixmob if age >=60 & age<=75 [pweight=poids_ind], vce(robust) irr compare**
